# UAV-based individual Chinese cabbage weight prediction using multi-temporal data

**DOI:** 10.1038/s41598-023-47431-y

**Published:** 2023-11-17

**Authors:** Andrés Aguilar-Ariza, Masanori Ishii, Toshio Miyazaki, Aika Saito, Hlaing Phyoe Khaing, Hnin Wint Phoo, Tomohiro Kondo, Toru Fujiwara, Wei Guo, Takehiro Kamiya

**Affiliations:** 1 Graduate School of Agricultural and Life Sciences, The University of Tokyo, 1-1-1, Yayoi, Bunkyo-ku, Tokyo, 113-8657 Japan; 2 Institute for Sustainable Agro-Ecosystem Services, Graduate School of Agricultural and Life Sciences, The University of Tokyo, 1-1-1, Midoricho, Nishitokyo-shi, Tokyo, 188-0002 Japan; 3 Nippon Norin Seed Co., 6-6-5 Takinogawa, Kita-ku, Tokyo, 114-0023 Japan

**Keywords:** High-throughput screening, Data acquisition, Machine learning

## Abstract

The use of unmanned aerial vehicles (UAVs) has facilitated crop canopy monitoring, enabling yield prediction by integrating regression models. However, the application of UAV-based data to individual-level harvest weight prediction is limited by the effectiveness of obtaining individual features. In this study, we propose a method that automatically detects and extracts multitemporal individual plant features derived from UAV-based data to predict harvest weight. We acquired data from an experimental field sown with 1196 Chinese cabbage plants, using two cameras (RGB and multi-spectral) mounted on UAVs. First, we used three RGB orthomosaic images and an object detection algorithm to detect more than 95% of the individual plants. Next, we used feature selection methods and five different multi-temporal resolutions to predict individual plant weights, achieving a coefficient of determination (R^2^) of 0.86 and a root mean square error (RMSE) of 436 g/plant. Furthermore, we achieved predictions with an R^2^ greater than 0.72 and an RMSE less than 560 g/plant up to 53 days prior to harvest. These results demonstrate the feasibility of accurately predicting individual Chinese cabbage harvest weight using UAV-based data and the efficacy of utilizing multi-temporal features to predict plant weight more than one month prior to harvest.

## Introduction

Vegetable production is often hindered by adverse environmental conditions, such as climate and weather variability^1,2^. To overcome these challenges and ensure stable production, genetic improvements through breeding^3^ and anticipatory crop-management corrections^1^ are needed. The implementation of these solutions requires the collection of phenotypic data throughout the growth cycle. However, this process is typically performed manually, which can be both costly and time-consuming, especially for crops that require individual plant characterization^4^.

Remote sensing data have become a popular alternative for crop phenotyping because they can provide information on a large scale in a non-expensive and non-destructive manner^5^. The rapid development of sensors and platforms has boosted remote sensing surveys using devices such as unmanned aerial vehicles (UAVs). Owing to their versatility in capturing data at different spatial and temporal resolutions^6,7^, features obtained from sensors such as visible-light (RGB) cameras, multi-spectral (MS) cameras, light detection and ranging systems (LiDAR), and thermal infrared imagery have been used for crop phenotyping^3,7^. Furthermore, in combination with image analysis techniques, additional features derived from the sensors are computed to represent the crop morphological and physiological attributes^4,8,9^. For example, techniques such as structure-from-motion (SfM), used for 3D point cloud reconstruction, and vegetation indices (VIs) computed from spectral reflectance imagery provide important insights into plant structure and nutritional conditions^10,11^. These multi-sourcing features, in combination with statistical and machine learning regression methods, have provided promising results in biomass prediction for crops such as coffee^12^, potatoes^13^, fava beans^14^, alfalfa^9^, soybeans^10^, wheat^15^, and cotton^16,17^.

Despite the advances in yield prediction using crop phenotyping with UAV-based data, these data are typically obtained at the canopy level rather than at the individual plant level^9,18^. However, this approach hinders the phenotyping of individually sold vegetables, such as Chinese cabbage, where pricing is determined on the basis of weight per individual plant rather than weight per unit area^4^. Although Chinese cabbage is an economically important crop in East Asia^19^, few studies have investigated the use of UAV-based phenotyping of individual plants. For example, Kang et al.^20^ used a multi-spectral camera to acquire data at a single time point to predict the weight of Chinese cabbage. Similarly, Kim et al.^21^ predicted multiple morphological attributes including fresh weight by extracting UAV-based data using bounding boxes. Although both studies predicted Chinese cabbage weight using either multi-spectral or RGB imagery, individual plant data were extracted by applying pixel-based segmentation to areas that were manually located to represent the plant location.

To efficiently obtain individual plant multi-temporal UAV-based data, we developed an approach to automatically detect individual Chinese cabbages in the field using an object detection algorithm known as YOLOv5^22^, and predicted individual plant weights using machine learning models. Furthermore, we evaluated multi-temporal features to predict Chinese cabbage weight several days prior to harvest. Our approach demonstrates the feasibility of predicting individual Chinese cabbage weights using UAV-based data up to 53 days before harvest with an RMSE of 560 g/plant and R^2^ = 0.72.

## Results

### UAV flights and weight measurement

The Chinese cabbage growth period in the field was 104 days, during which the UAV collected data from 26 different time points (TPs) using two cameras (RGB and MS) (Table S1). During each flight, 67 and 133 images were captured using the RGB and MS cameras, respectively.

In total 1196 plants that were planted in the field, we measured the weight of 872 plants; the remaining plants were not included, mainly because of workforce limitations. The average plant weight measured was 2847 ± 940 g/plant. Weight variability can be attributed to differences in the genotype of the F2 population.

### Individual plant detection

To save time in detecting individual plants, we applied an object-detection algorithm instead of manually drawing a bounding box for each plant. We used an object-detection algorithm based on convolutional neural networks (YOLO) to automatically locate individual Chinese cabbage plants. To evaluate the accuracy of the detection algorithm, the field was divided into two groups: training (n = 589) and testing (n = 547). Once the model was trained, it was applied to the test dataset. The model detected 469 (85.7%), 528 (96.5%), and 539 (98.5%) plants on September 29, October 4, and October 6, respectively (Fig. S1). The lowest accuracy (85.7%) was obtained for the first date (12 DAT).

**Figure 1:**
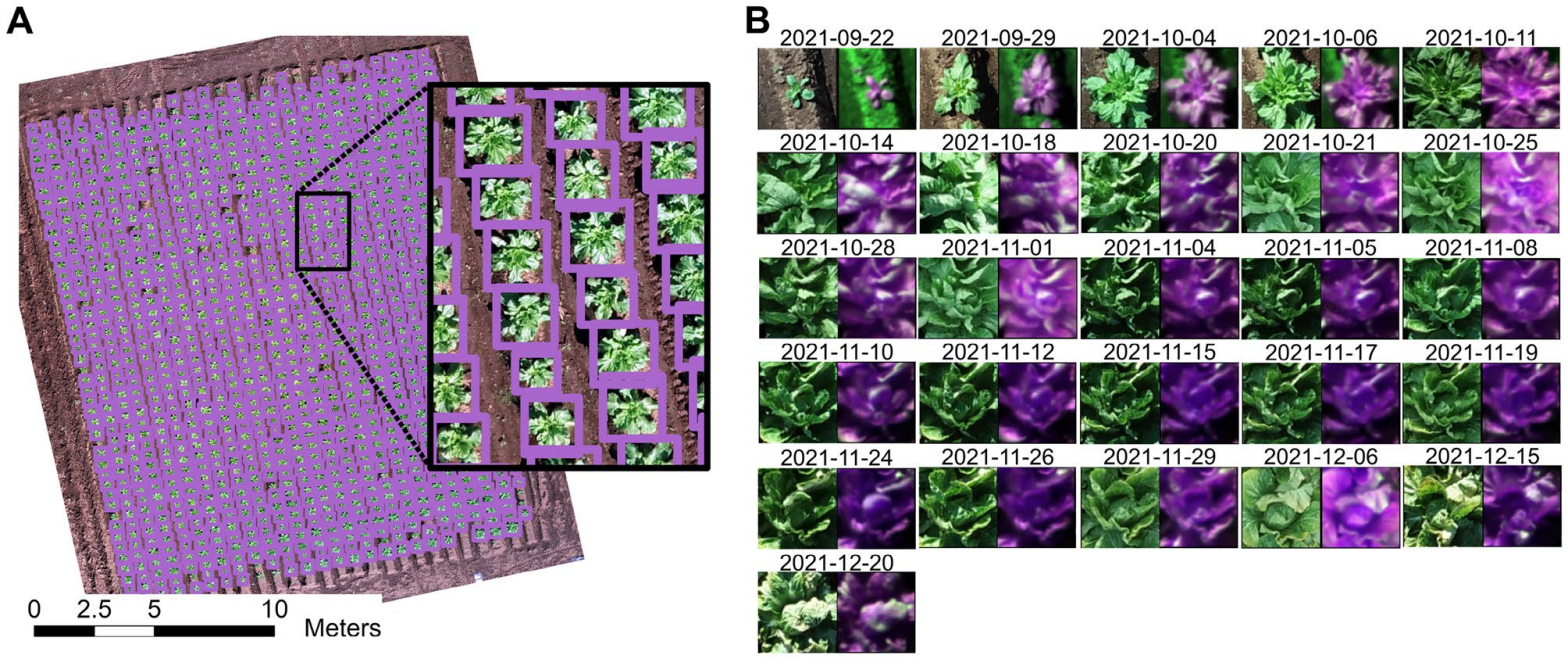
Individual plant detection. (**A**) The final bounding boxes (purple) were obtained by merging the bounding boxes for three TPs (September 29, October 4, and October 6, 2021). The black box is an enlarged area as an example of plants detected by the model. Figure prepared using ArcGIS Pro version 3.0^24^ (**B**) An example of the multi-temporal RGB and MS imagery extracted for an individual plant. On the left is the true-color RGB image, while on the right is the false-color MS image (NIR—red-edge—red). The individual plant RGB and MS imagery were obtained with the Matplotlib package in Python version 3.9^25,26^.

Then, we merged the bounding boxes (see the “Materials and methods” section). This enabled us to obtain the final individual plant bounding box. A total of 1136 individual plants were detected, representing 95% of the total number of plants (Fig. 1A). Plants that were not detected had smaller leaf diameters than other plants (Fig. 1A; Fig. S1). Finally, to extract information from the RGB and MS orthomosaic images (Fig. 1B), we transformed the bounding box coordinate system from the image spatial reference to the projected coordinate system (UTM zone 54N), in which the UAV captured the data.

### Search for the most relevant features for individual plant weight prediction

In total, 18 features were computed for each identified plant across 26 TPs. These features were grouped into three levels: the first level included eight features derived from the RGB and MS orthomosaic imagery, the second level included seven VIs, and the third level encompassed three features derived from the 3D point cloud data. We reduced the number of features to reduce multicollinearity. Three feature selection methods were used: two based on wrapper methods [exhaustive search and sequential feature selection (SFS)] and one based on an embedded method [random forest (RF)]^27^. The most recent TP data (December 20, 2021) were used to train each filtering method. Out of 872 plants weighed, 33 plants were not detected by YOLOv5 and therefore 839 plants were used for the weight prediction. We split them into two datasets: a training dataset (n = 756) and a test dataset (n = 83).

**Figure 2:**
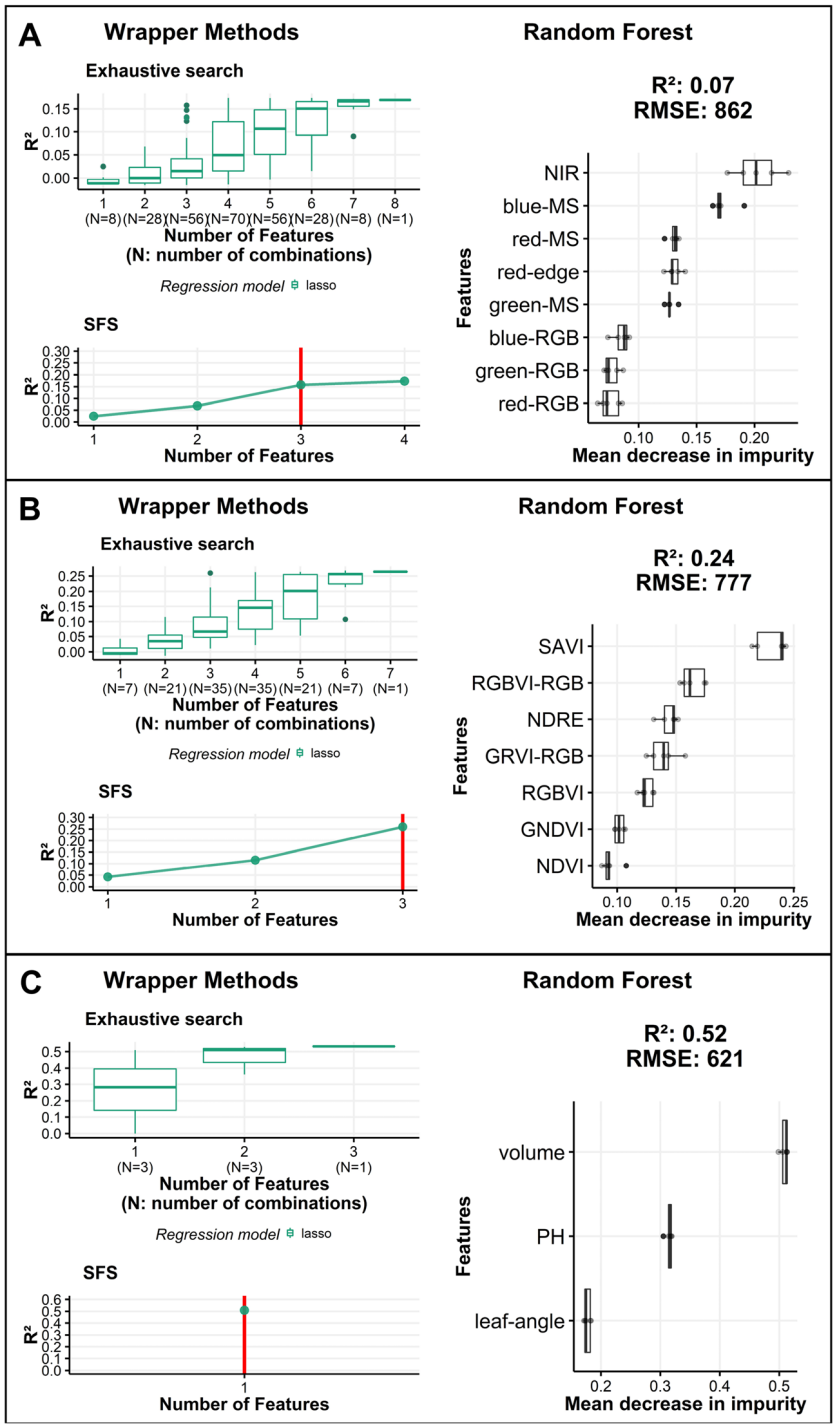
Wrapper [exhaustive research and sequential feature selection (SFS)] and embedded [random forest (RF)] method results for three levels of information. (**A**) First level: images taken directly with RGB and MS cameras. (**B**) Second level: vegetation indices (VIs). (**C**) Third level: features derived from the 3D point cloud data. The red line in the SFS plots represents the number of selected features. Box plot and bar plot figures were created with the ggplot2 package in R version 4.1.1.^28,29^.

For the first level, the exhaustive search and SFS methods yielded similar accuracies (R^2^ = 0.16) to those using more features. For example, the incorporation of four features resulted in the highest R^2^ of 0.17 (Fig. 2A). As no significant improvement incurred by including more than three features, we selected features that occupied the top three positions in the RF importance index [Near Infrared (NIR), blue-MS, and red-MS]. For the second level, when the wrapper method was applied to the seven VIs, the models trained with three VIs yielded the highest accuracy (R^2^ = 0.26) (Fig. 2B). Although the top three features ranked by the RF were SAVI, RGBVI-RGB, and NDRE, both wrapper methods yielded the highest accuracy using SAVI, RGBVI-RGB, and GRVI-RGB. Therefore, these three features were selected for this study. Finally, for the third level, the exhaustive search indicated that by using one variable (volume), the models achieved an R^2^ of 0.51, and by including all three variables, the models achieved an R^2^ of 0.53 (Fig. 2C). Similarly, the SFS and RF indicated that volume was the most relevant feature for predicting weight. Therefore, volume was selected as the feature for the third level. In summary, for individual plant weight prediction, seven features were selected: three from the first level (red-MS, blue-MS, and NIR), three from the second level (SAVI, RGB-RGB, and GRVI-RGB), and one from the third level (volume).

### Assessing the selected features with five regression models

**Figure 3:**
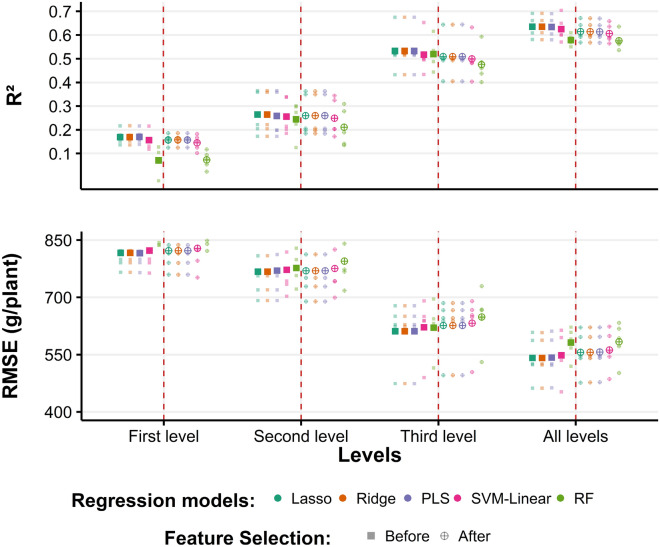
Comparison of the regression model predictions, before and after applying the feature selection methods on three levels of information (RGB and MS image layers, vegetation indices, and features derived from the 3D point cloud data) and combining all levels. The small dots represent each of the five-fold cross-validation results. The red dotted line separates the evaluation metrics before (left) and after (right) feature selection. The colors indicate each regression model [lasso, ridge, PLS, SVM-linear, random forest (RF)]. The small and large points represent the result of five-fold cross-validation and the mean value of the cross-validation results, respectively. Dot plots were created with the ggplot2 package in R version 4.1.1.^28,29^.

To evaluate the feature selection results, we trained five regression models: partial least squares (PLS), lasso, ridge, support vector machine (SVM-linear), and RF. We used the last TP data to train the models with the five-fold cross-validation datasets, which were the same as those used for feature selection. For each level of information, we trained the regression models using all the features (Fig. 3) or selected features (Fig. 3). For example, at the first level, there were eight features before selection (Table S2) and three features (red-MS, blue-MS, and NIR) after selection. In addition, we trained the regression models using all 18 features and the seven selected features (all levels in Fig. 3).

The best model performance was achieved when all levels of information were combined (R^2^ = 0.64; RMSE = 541 g/plant) (Fig. 3. Before feature selection using Ridge), whereas the worst prediction was obtained by the models that only used features from the first level (Fig. 3). Although the model with the best accuracy was the one trained with all 18 features as input, the models trained with the seven selected features achieved similar accuracies (R^2^ = 0.62; RMSE = 556 g/plant) (Fig. 3. After feature selection using PLS). These results indicate that, regardless of the reduction in the number of features, the models did not lose their prediction capability. The RF models exhibited the lowest performance at all levels (represented in green in Fig. 3).

### Weight prediction using multi-temporal data

The use of multi-temporal data for weight prediction was evaluated using the following two questions: (1) Does weight prediction improve when multi-temporal data are involved? (2) What is the optimal UAV multi-temporal resolution for weight estimation? To answer these questions, we evaluated the values predicted by the regression models using five-fold cross-validation and a test dataset. The models were trained using the seven selected features computed for the 26 TPs.

**Figure 4:**
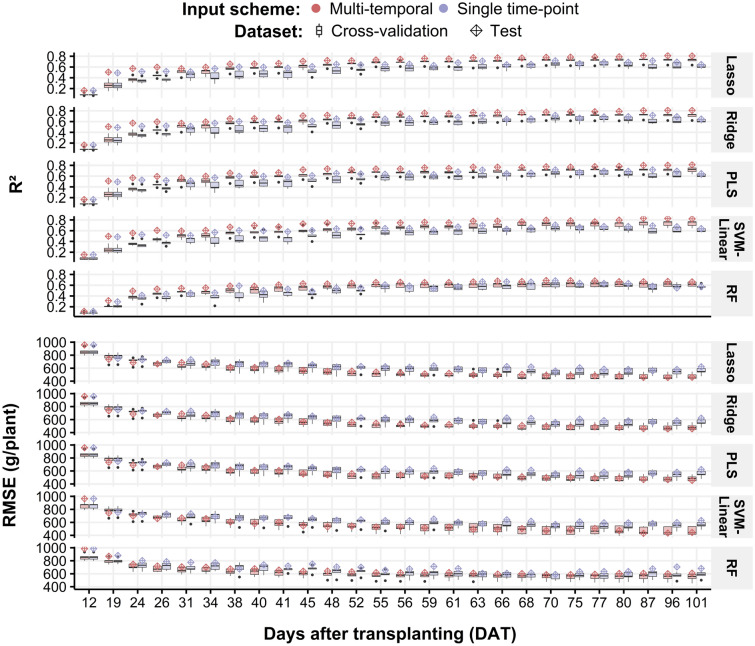
R^2^ (top) and RMSE (bottom) of the five-fold cross-validation and test results for each regression model [lasso, ridge, PLS, SVM-linear, and random forest (RF)]. The results were compared for two input schemes: multi-temporal (red) and single-time-point (purple) data. The boxplots show a five-fold cross-validation results distribution, whereas the diamond points indicate the test dataset results. Box plots were created with the ggplot2 package in R version 4.1.1.^28,29^.

For the first question, we separately trained the regression models with features extracted from two temporal schemes: single- and multi-temporal points. The single time-point scheme was computed for a specific TP, whereas the multi-temporal point scheme used not only specific TP data but also sequencing data from the first TP (12 DAT). We used five-fold cross-validation and test datasets to compare the weight predictions using both schemes. The best average cross-validation accuracy was obtained by the SVM-linear model using multi-temporal data between 12 and 96 DAT (R^2^ = 0.73; RMSE = 464 g/plant); for the single time-point scheme, the best cross-validation results were obtained by the PLS model using data at 77 DAT (R^2^ = 0. 66; RMSE = 525 g/plant) (Fig. 4). For the test dataset, the SVM-linear model trained with a multi-temporal scheme yielded the best accuracy using data up to 96 DAT (R^2^ = 0. 83; RMSE = 436 g/plant), whereas the lasso model achieved the best prediction accuracy for the single time-point scheme using data at 70 DAT (R^2^ = 0. 72; RMSE = 557 g/plant) (Fig. 4). These results show that when multi-temporal data were used to train the models, the accuracy of the weight prediction improved.

**Figure 5:**
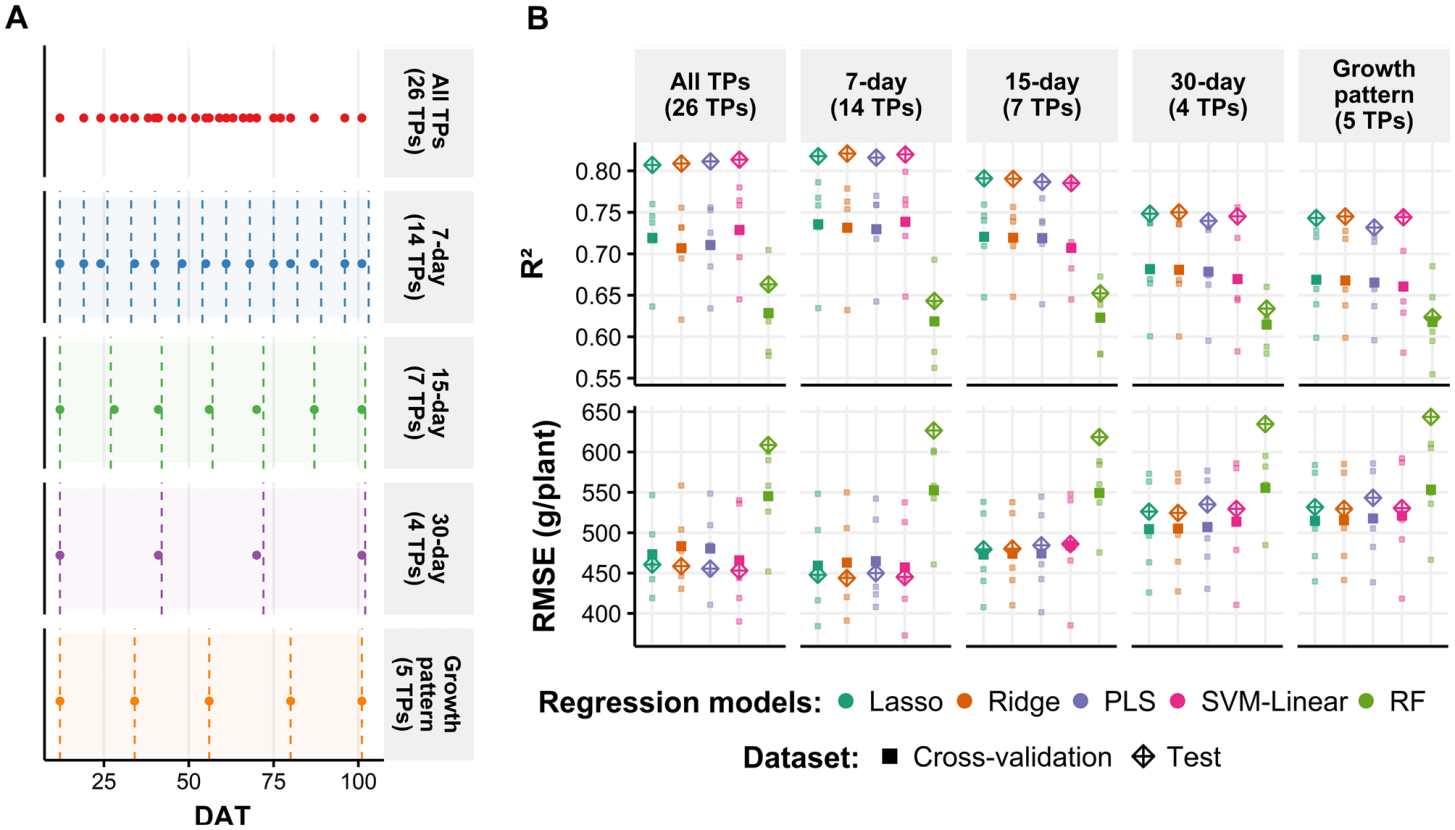
Individual plant weight prediction using 5 different multi-temporal resolutions. (**A**) The panel shows the selected TPs in each multi-temporal resolution. (**B**) The R^2^ and RMSE five-fold cross-validation and test results for 5 different multi-temporal resolutions. The colors indicate each regression model [lasso, ridge, PLS, SVM-linear, random forest (RF)]. The small and large square points represent the result of five-fold cross-validation and the mean value of the cross-validation results, respectively. The diamond points indicate the test dataset results. Plots were created with the ggplot2 package in R version 4.1.1.^28,29^.

For the second question, we used five multi-temporal resolutions (Fig. 5A). Four resolutions include a reduced number of TPs and one resolution was represented by all 26 TPs (“All TPs” in Fig. 5A). To reduce the number of TPs, three multi-temporal resolutions were set based on fixed time intervals (7-, 15-, and 30-day intervals) and one resolution (growth pattern) was set based on the Chinese cabbage growth cycle (12, 34, 56, 80, and 101 DAT; Fig. S2). As a result, at each multi-temporal resolution, 26, 14, 7, 4, and 5 TPs were used for all TPs, 7-day, 15-day, 30-day, and growth patterns, respectively (Fig. 5A). To compare the prediction accuracy obtained among the five multi-temporal resolutions, we used the fivefold cross-validation and test datasets. The best averaged cross-validation accuracy prediction was obtained by the 7-day multi-temporal resolution using data from 14 TPs (R^2^ = 0.74; RMSE = 457 g/plant), and a similar accuracy was obtained when the models were trained using all 26 TPs (R^2^ = 0.73; RMSE = 466 g/plant) and seven TPs (R^2^ = 0.72; RMSE = 473 g/plant) (Fig. 5B). For the test dataset, the best accuracy was obtained by the 7-day multi-temporal resolution (R^2^ = 0.82; RMSE = 444 g/plant), followed by all 26 TPs (R^2^ = 0.81; RMSE = 444 g/plant) (Fig. 5B) and 15-day (7 TPs) resolutions (R^2^ = 0.79; RMSE = 479 g/plant). These results indicate that regardless of the reduction in the number of TPs from 26 to 14 and 7, the regression models can still achieve reliable predictions.

### Weight prediction prior to harvest

**Figure 6:**
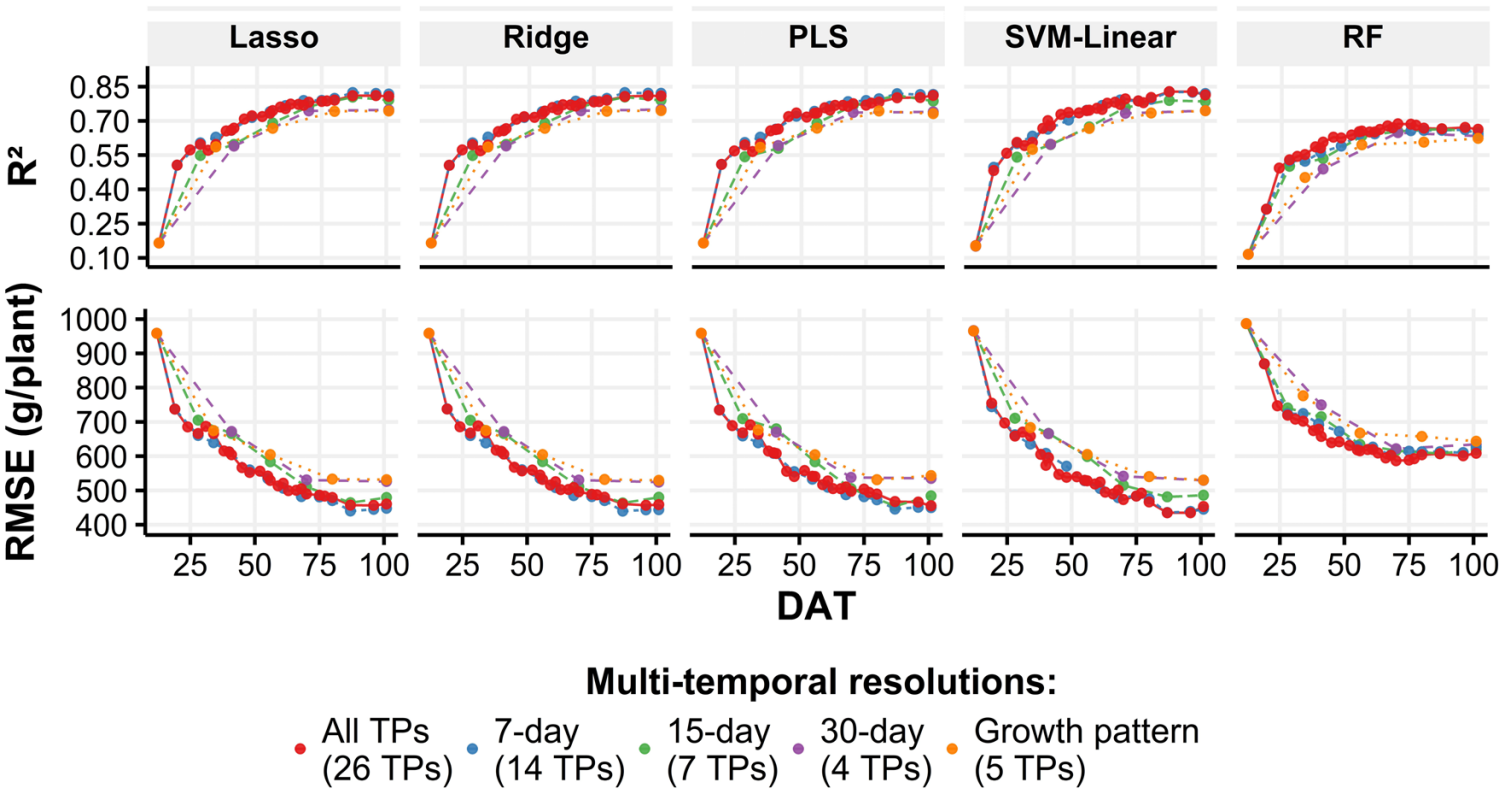
Early individual plant weight prediction. Prediction results (R^2^ and RMSE) obtained from the test dataset (n = 83). The dots represent the results yielded by the regression models when these were trained using sequenced TPs data from 12 DAT up to the dot position. Each multi-temporal resolution is represented by different colors. Plots were created with the ggplot2 package in R version 4.1.1.^28,29^.

To assess the ability to predict Chinese cabbage weight before harvest, we trained regression models using multiple sequences of TP data. For example, to predict Chinese cabbage weight using data up to 30 days prior to harvest (DPH), we used 20, 9, 4, 3, and 3 TP data points for each of the five multi-temporal resolutions (all TPs, 7-day, 15-day, 30-day, and growth patterns). The weight prediction was performed using the test dataset. The best prediction accuracy (R^2^ = 0.83; RMSE = 436 g/plant) was obtained by the SVM-linear model using the sequence TP data between the transplantation date (12 DAT) and 5 DPH (96 DAT) for all TP multi-temporal resolutions (Fig. 6, red lines). For the other multi-temporal resolutions, the 7-day resolution achieved the highest prediction accuracy (R^2^ = 0.83; RMSE = 453 g/plant) when the model used 12 TP-sequenced data up to 14 DPH (87 DAT) (Fig. 6, blue lines). In addition, the 15-day interval models yielded an R^2^ = 0.81 and RMSE = 455 g/plant using 6 TP (87 DAT, 14 DPH) (Fig. 6, green lines). Furthermore, the models trained with sequenced data up to 53 DPH yielded R^2^ greater than 0.72 and RMSE less than 560 g/plant (Fig. 6, in PLS). This was achieved using 7-day and all TP multi-temporal resolutions. At 15 days, the earliest date to obtain predictions with R^2^ greater than 0.72 (R^2^ = 0.77; RMSE = 505 g/plant) was 31 DPH (Fig. 6).

## Discussion

In previous studies, UAV-based data extraction to characterize Chinese cabbage plants was based on either manually drawing a bounding box or using pixel-based classification methods^20,21,30^. Although these methods are useful for obtaining crop phenotyping features at the canopy level, they hinder a more precise and efficient understanding at the individual plant level^4,31^. In this study, an object detection algorithm automatically detects 95% of the Chinese cabbage plants sown in the field. In addition, the predicted bounding boxes facilitated the extraction of individual plant images captured by the two cameras (RGB and MS). Furthermore, individual plant images facilitated the computation of features, grouped into three levels (RGB-MS, VI, and 3D point-cloud data), throughout multiple TPs.

In this study, the weight prediction accuracy was evaluated under different combinations of UAV-derived features and multi-temporal resolutions. The most relevant features for predicting Chinese cabbage weight were identified from the three levels of UAV information. Regression models trained with a reduced number of UAV-derived features (seven) achieved accuracy predictions similar to those trained with all features (18) (Fig. 2), showing that using a large number of inputs for training is not efficient^12^. Moreover, the results indicated that features from 3D point-cloud data, such as volume, achieved the highest prediction accuracy (Fig. 2), confirming the importance of including 3D-derived features for crop biomass prediction^14,32^. Furthermore, combining UAV information from multiple sources (RGB, MS, VIs, and 3D point cloud) resulted in an increase in weight prediction accuracy of 0.11 in R^2^ and a decrease of 70 g/plant in RMSE compared to the one using only volume. These results indicated the effectiveness of using UAV information from multiple sources^10,14^.

To optimize the number of TPs required for weight prediction, two issues were evaluated: the advantage of using multi-temporal data over a single time-point prediction and the prediction performance of regression models trained with lower multi-temporal resolution. For the first issue, implementing multi-temporal data led to an increase in the weight prediction accuracy in the test dataset in R^2^ (0.17) and a decrease in RMSE (178 g/plant) compared to using only single time-point features (Fig. 4). This confirms previous findings on the use of multi-temporal remote sensing data for crop biomass prediction^14,18^. Secondly, models using a lower multi-temporal resolution (14 TPs) yielded higher accuracies than those trained with all 26 TPs (Fig. 5). These results indicate that lower multi-temporal resolution (7-day and 15-day ones) can provide sufficient information for monitoring Chinese cabbage crops.

In addition to identifying the most relevant UAV features and multi-temporal resolutions for predicting Chinese cabbage weight, this study addressed the capability of predicting individual plant weights prior to harvest. By using sequenced TP data up to 53 DPH, the models started to achieve accuracies in R^2^ greater than 0.72 and RMSE lower than 560 g/plant, reaching R^2^ values of 0.8 and RMSE of 467 g/plant when data up to 21 DPH were used. This early prediction of weight can be explained by the fact that the weight of Chinese cabbage is linearly proportional to plant height^21^. The final plant size and shape were determined after the rosette stage^33^ (Fig. S2). Our results are comparable to those reported by Kim et al.^21^ for a test dataset with R^2^ values greater than 0.76. However, unlike their approach of predicting weight using RGB information at the time of harvest, our multi-temporal UAV-based data allowed us to predict weight with R^2^ > 0.8 at 21 DPH. These results indicate that the use of multi-sourcing and multi-temporal UAV-based data significantly improves the accuracy of weight prediction and enables early weight prediction.

## Conclusions

In this study, we explored the feasibility of predicting individual plant weights by automatically generating and optimizing multi-temporal UAV-based data. The proposed methodology integrates an object detection model that identifies plants within a field and computes the features from two cameras (RGB and MS) at the plant level. Furthermore, the effective use of relevant multi-temporal features to predict biomass enabled the prediction of Chinese cabbage weight at 53 DPH. The ability to predict early weight is important for farmers and breeders in crop management.

## Materials and methods

**Figure 7:**
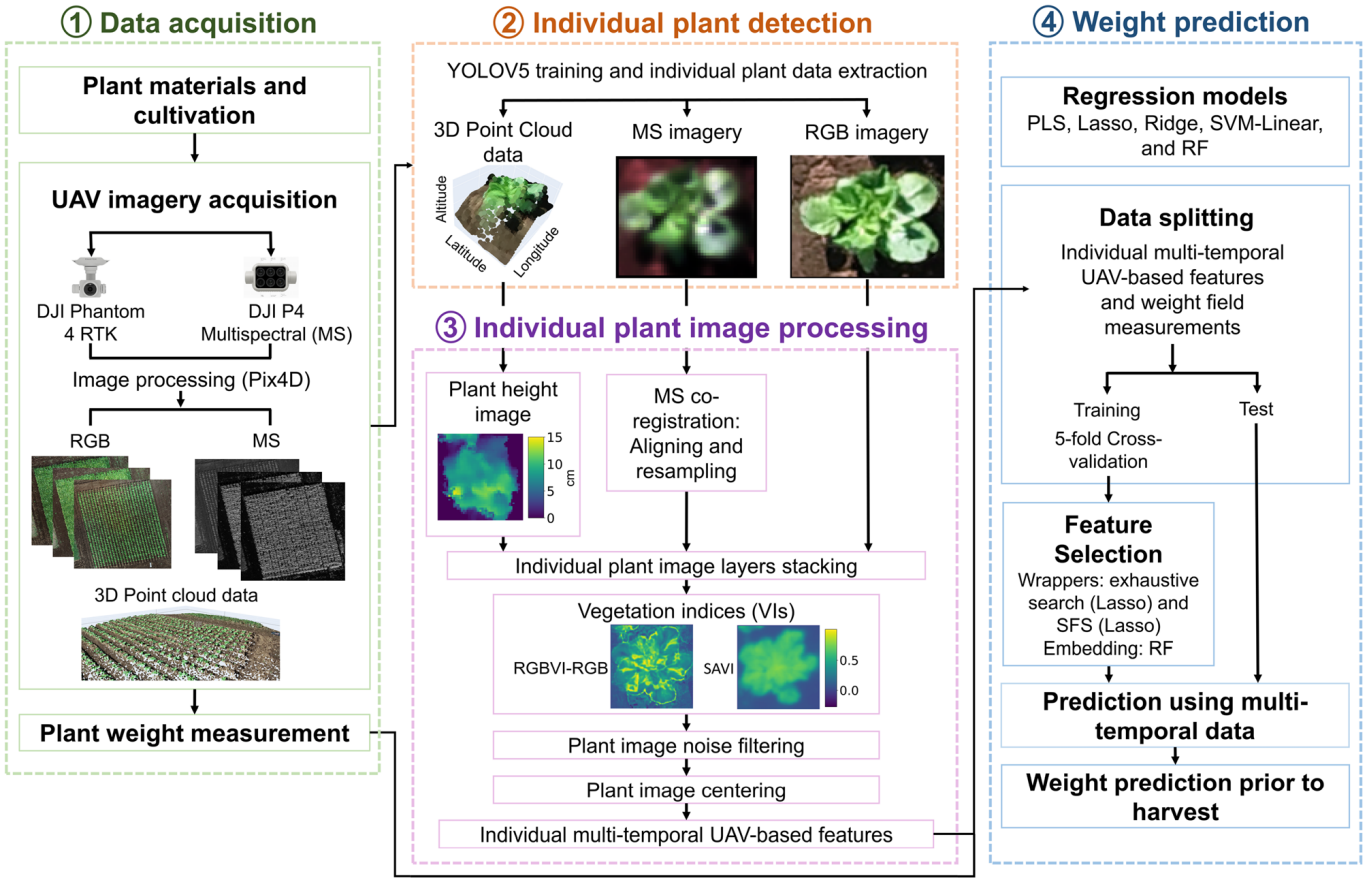
Workflow of the proposed methodology that uses the most relevant RGB, MS, and point cloud data to predict individual Chinese cabbage plant weight prior to harvest. The general framework is divided into four stages: data acquisition, individual plant detection, individual plant image processing, and weight prediction. Individual plant images obtained with Matplotlib package in Python version 3.9^25,26^.

The pipeline implemented in this study was divided into four main steps, as shown in Fig. 7.

### Data acquisition

Plant materials and cultivation: Chinese cabbage cultivars were purchased, and their genetically fixed lines (P1 and P2) as well as their F2 populations were cultivated in a field at the Institute for Sustainable Agro-Ecosystem Services at the University of Tokyo, Nishi-Tokyo, Tokyo, Japan. Experimental research and field studies on plants, including the collection of plant materials, comply with relevant institutional, national, and international guidelines and legislation.

UAV imagery acquisition: The image-capturing process was conducted from September 22 to December 20, 2021, in an experimental field located in Tokyo, Japan. On each capture date, two UAVs were flown: a DJI Phantom 4 RTK and a DJI P4 Multispectral (SZ DJI Technology Co., Shenzhen, China). The DJI Phantom 4 RTK captured RGB images with a resolution of 5472 × 3648 pixels. The P4 Multispectral (MS) obtained imagery in five different bands: blue (450 nm ± 16 nm), green (560 nm ± 16 nm), red (650 nm ± 16 nm), red edge (730 nm ± 16 nm), and near-infrared (NIR) (840 nm ± 26 nm), with a pixel resolution of 1600 × 1300. Additionally, the flight altitude and image ratio overlap (front and side) were set to 15 m and 80%, respectively. To improve spatial accuracy, both UAVs were connected to a network RTK Service (“ichimill,” SoftBank Corp., Tokyo, Japan). Four ground control points (GCPs) were placed at the field corners. Images were processed using the photogrammetric software Pix4D Mapper (version 4.6; Pix4D SA, Prilly, Switzerland)^23^. This software was applied to create RGB and MS orthomosaic imagery and generate 3D point cloud data (Fig. 7). The configuration for Pix4D processing was set as described by Want et al.^34^, as proposed in their study.

The individual plant weight was measured after 102 and 103 days after transplanting. The plants were cut off from the base and fresh weight was measured using a balance. To avoid errors in weighing, the plants were cut and placed in the order in which they were planted in the field and then weighed. This procedure was repeated for each row (46 plants/row) to ensure that the number of plants was accurate.

### Individual plant detection

Individual Chinese cabbage plants were detected using YOLOv5 implemented in PyTorch^35^. To facilitate labelling and model training, the RGB orthomosaic for each TP (September 29, October 4, and October 6) was divided into 512 × 512 tiles. For labelling, the tiles belonging to the training dataset were uploaded to the RoboFlow platform^36^, where the bounding boxes were manually drawn. Using RGB orthomosaic imagery as a reference, we manually drew bounding boxes for 975 plants across the three TPs. To augment the training dataset, three transformation functions were randomly applied to each tile: rotation, hue-saturation-brightness modification, and expansion (Fig. S3). These functions were executed using the OpenCV-Python package^37^.

To obtain a unique bounding box for each plant, the predicted bounding boxes obtained for each TP RGB orthomosaic image were merged. For this, bounding boxes whose intersected areas were greater than 40% were found and grouped. Subsequently, each group of intersecting bounding boxes was merged into a single bounding box by selecting the maximum height and width that covered all boxes. The merging step was performed using the GeoPandas library in Python^38^.

To assign each plant data to its corresponding bounding box, we created a spatial point grid geometry using QGIS^39^, with a spacing of 45 × 65 cm (distance between plants and rows). Each spatial point contained a unique index for plant identification. The spatial distance (SPd) from the point center (Pc) to every neighboring bounding box (BBc) was computed using Euclidean distance equation (Eq. 1). The closest bounding box to the spatial point with a distance less than 45 cm (plant distance sowing) was indexed to the point information.

1 $${\text{SPd}} = \sqrt {\left( {Pc_{x} - BBc_{x} } \right)^{2} + \left( {Pc_{y} - BBc_{y} } \right)^{2} }$$

The object detection performance was measured using the overall accuracy of the test field^40^.

### Individual plant image processing

To compute the features of each plant across the 26 TPs, the data derived from the RGB, MS, and 3D point clouds created by the RGB camera were processed, as shown in Fig. 7.

Plant-height image: To transform the 3D point cloud into a plant height image, the 3D point cloud was interpolated using neighboring values. To obtain an accurate plant height value, the reference surface value was subtracted from each plant-height image. The surface value was obtained from the first TP data (September 22, 2021) because the soil surface was not fully covered by plants. The spatial accuracy of the 3D point cloud data in the X- and Y- dimensions was assessed by comparing two RGB images: RGB imagery taken by RGB camera and 2D RGB image from 3D point cloud data. Two metrics were used: cross-correlation displacement^41^and correlation-coefficient. Cross-correlation is a similarity metric, quantifying the displacement between the two images transformed into Fourier space. The assessment was conducted on a randomly selected 100 plants. For each plant, two types of RGB images (RGB camera and 2D RGB image from 3D point cloud data) were transformed into grayscale images. (Fig. S4). The maximum displacement in X and Y was 3.81 mm; the median of correlation coefficient was 0.89.

MS co-registration: To use both the RGB and MS imagery, the MS imagery was registered to the RGB resolution. Registration of the MS imagery was divided into two steps: alignment and resampling. The alignment step was performed using phase cross-correlation. This determines the linear shift of the two images by obtaining a correlation peak in their frequency domain representation, which is obtained by applying an inverse Fourier transform^41,42^. MS resampling of the RGB spatial resolution was computed using a k-nearest neighbor interpolation.

Individual plant image layer stacking: To facilitate calculations across the multi-source image layers for all TPs, the three image sources (RGB, MS, and plant height images) were stacked into an individual plant image with four dimensions (TPs, layers, longitude, and latitude) using the Xarray package in Python^26,43^.

Vegetation indices (VIs): Once the individual plant images were obtained, we calculated the VIs using the equations^44–50^ listed in Table 1. Initially, 13 VIs were calculated, but this number was reduced to seven after excluding highly correlated VIs (Pearson correlation coefficient > 0.95) (Fig. S5, red boxes).

**Table 1:** The computed vegetation indices.

Vegetation Indices (VIs)	Formula	References	Camera
**GRVI**: Green and red ratio vegetation index	$\frac{{{\text{green}} - {\text{red}}}}{{{\text{green}} + {\text{red}}}}$	^44^	RGB and MS
**MGRVI**: Modified green–red vegetation index	$\frac{{{\text{green}}^{2} - {\text{red}}^{2} }}{{{\text{green}}^{2} + {\text{red}}^{2} }}$	^45^	RGB and MS
**RGBVI**: Red–green–blue vegetation index	$\frac{{{\text{green}}^{2} - \left( {\text{blue*red}} \right)}}{{{\text{green}}^{2} + \left( {\text{blue*red}} \right)}}$	^45^	RGB and MS
**GNDVI**: Green normalized difference vegetation index	$\frac{{NIR - {\text{green}}}}{{NIR + {\text{green}}}}$	^46^	MS
**NDRE:** Normalized difference red-edge index	$\frac{{NIR - {\text{red}}\_{\text{edge}}}}{{NIR + {\text{red}}\_{\text{edge }}}}$	^47^	MS
**NDVI:** Normalized difference vegetation index	$\frac{{NIR - {\text{red}}}}{{NIR + {\text{red}}}}$	^48^	MS
**RECI:** Red-edge chlorophyll index	$\frac{NIR}{{{\text{red}}\_{\text{edge }}}} - 1$	^49^	MS
**SAVI**: Soil-adjusted vegetation index	$\frac{{NIR - {\text{red}}}}{{NIR + {\text{red}} + 0.5}}{*}1.5$	^50^	MS

Blue, green, red, near-infrared (NIR), and red-edge represent the image layer values.

Plant image noise filtering: Three filters were applied to each individual plant image to remove and reduce the noise caused by non-vegetation (soil) pixels, contours that were not neighboring the main plant, and pixel values affected by brightness and shadows (Fig. S6). The first two filters were applied to all the image layers in the individual plant image, whereas the third filter was applied only to the RGB layers. For the first filter, a soil mask was obtained from a k-means model trained using seven VIs. The k-means model was implemented using the scikit-learn package. To implement the second filter, the OpenCV-Python package^37^ was used to compute contours wrapping neighboring pixels, and a filter based on their area was then applied to remove smaller contours. For the third filter, a histogram equalization technique^51^ was applied to each RGB layer. A histogram was constructed using the Numpy package^52^.

Plant image centering: To center the individual plant images, a convex hull polygon was computed using the first TP RGB plant image (September 22, 2021). The geometric center of the polygon was obtained by averaging its vertices, and each plant image layer was then shifted to the center of the polygon. The SciPy package was used to obtain convex hulls^53^. Finally, the image was reduced by 70% from the center to the border (Fig. S6).

Individual multi-temporal UAV-based features: Individual plant image layers were summarized as features. The RGB and MS layers were summarized using the median statistic (Fig. S7) and the plant height image was transformed into three features: plant height (PH), leaf angle, and volume. The PH of each TP was computed as the 90th percentile of plant height image values (Fig. S8). The leaf angle was calculated as the angle between each pixel vector (${P}_{p}$) (represented as longitude, latitude, and altitude) and a normal vector located in the plant height image center (${V}_{c}$); the distance between the two vectors was used to compute the angle (Eq. 2). The individual plant volume was calculated as the sum of all pixel height values (${H}_{i}$), which comprised the 2D plant height image, and was then multiplied by the pixel area in cm^2^ (${a}_{p}$) (Eq. 3)^4^.

2 $$\cos \left( {leaf\;angle} \right) = { }\frac{{P_{p} { }V_{c} { }}}{{\left\| {P_{p} } \right\|\left\| {V_{c} } \right\|}}$$

3 $${\text{Volume}} = { }\mathop \sum \limits_{i = 1}^{n} H_{i} {*}a_{p} { }$$

Finally, to train the regression models, the features were standardized using the StandardScaler function from the scikit-learn library^54^.

### Weight prediction

Regression models: Model hyperparameters were set through an exhaustive search using a five-fold cross-validation approach. The hyperparameters used to determine the optimal configuration for each model are listed in Table S3. Scikit-learn was used to implement and optimize the models.

Data splitting: To evaluate the weight prediction results, individual plant data were split into two groups: training (90%) and testing (10%) datasets. The training dataset was split into 5-folds to implement cross-validation (Fig. 7).

Feature selection: The lasso regression model was used to find the best input combination for both wrapper methods. For an exhaustive search, the total number of combinations was configured according to the total number of features available at each level of information. SFS was implemented using the Sequential Feature Selector function available in the scikit-learn library, and forward selection was set as the search method. The embedding method was applied through the RF, in which the mean decrease in impurity (MDI) was used to rank the feature importance.

### Evaluation metrics

To assess the accuracy of individual weight predictions, two evaluation metrics were implemented: the coefficient of determination (R^2^) (Eq. 4) and the root mean square error (RMSE) (Eq. 5).

4 $${\text{R}}^{{2}} = 1 - {\raise0.7ex\hbox{${\mathop \sum \nolimits_{i = 1}^{n} \left( {y_{i} - \hat{y}_{i} } \right)^{2} }$} \!\mathord{\left/ {\vphantom {{\mathop \sum \nolimits_{i = 1}^{n} \left( {y_{i} - \hat{y}_{i} } \right)^{2} } {\mathop \sum \nolimits_{i = 1}^{n} \left( {y_{i} - \overline{y}} \right)^{2} }}}\right.\kern-0pt} \!\lower0.7ex\hbox{${\mathop \sum \nolimits_{i = 1}^{n} \left( {y_{i} - \overline{y}} \right)^{2} }$}}$$

5 $${\text{RMSE}} = \sqrt {{ }\mathop \sum \limits_{i = 1}^{n} \left( {y_{i} - \hat{y}_{i} } \right)^{2} }$$

where, ${y}_{i}$ is the individual plant weight, $\overline{y }$ is the average weight, and ${\widehat{y}}_{i}$ is the individual plant weight predicted using the model.

### Supplementary Information

Supplementary Information.

## Data Availability

The UAV imagery generated and/or analyzed during the current study is available from the corresponding author upon reasonable request.

## Code availability

All code associated with the current study is available at: https://github.com/anaguilarar/CC_Weight_Prediction.

## References

[1] Bisbis MB, Gruda N, Blanke M (2018). Potential impacts of climate change on vegetable production and product quality—a review. J. Clean. Prod..

[2] Ray DK, Gerber JS, MacDonald GK, West PC (2015). Climate variation explains a third of global crop yield variability. Nat. Commun..

[3] Song P, Wang J, Guo X, Yang W, Zhao C (2021). High-throughput phenotyping: Breaking through the bottleneck in future crop breeding. Crop J..

[4] Guo W, Fukano Y, Noshita K, Ninomiya S (2020). Field-based individual plant phenotyping of herbaceous species by unmanned aerial vehicle. Ecol. Evol..

[5] Deng L (2018). UAV-based multispectral remote sensing for precision agriculture: A comparison between different cameras. ISPRS J. Photogram. Remote Sens..

[6] Yang G (2017). Unmanned aerial vehicle remote sensing for field-based crop phenotyping: Current status and perspectives. Front. Plant Sci..

[7] Guo W (2021). UAS-based plant phenotyping for research and breeding applications. Plant Phenom..

[8] Bannari A, Morin D, Bonn F, Huete AR (1995). A review of vegetation indices. Remote Sens. Rev..

[9] Tang Z (2021). Validation of UAV-based alfalfa biomass predictability using photogrammetry with fully automatic plot segmentation. Sci. Rep..

[10] Maimaitijiang M (2020). Soybean yield prediction from UAV using multimodal data fusion and deep learning. Remote Sens. Environ..

[11] Ghamisi P (2019). Multisource and multitemporal data fusion in remote sensing: A comprehensive review of the state of the art. IEEE Geosci. Remote Sens. Mag..

[12] Barbosa BDS (2021). UAV-based coffee yield prediction utilizing feature selection and deep learning. Smart Agric. Technol..

[13] Li B (2020). Above-ground biomass estimation and yield prediction in potato by using UAV-based RGB and hyperspectral imaging. ISPRS J. Photogram. Remote Sens..

[14] Ji Y (2022). Estimation of plant height and yield based on UAV imagery in faba bean (*Vicia faba* L.). Plant Methods.

[15] Fei S (2022). UAV-based multi-sensor data fusion and machine learning algorithm for yield prediction in wheat. Precis. Agric..

[16] Feng A, Zhou J, Vories ED, Sudduth KA, Zhang M (2020). Yield estimation in cotton using UAV-based multi-sensor imagery. Biosyst. Eng..

[17] Ashapure A (2020). Developing a machine learning based cotton yield estimation framework using multi-temporal UAS data. ISPRS J. Photogram. Remote Sens..

[18] Nevavuori P, Narra N, Linna P, Lipping T (2020). Crop yield prediction using multitemporal UAV data and spatio-temporal deep learning models. Remote Sens. Basel.

[19] Sun XX (2018). Genetic analysis of Chinese cabbage reveals correlation between rosette leaf and leafy head variation. Front. Plant Sci..

[20] Kang YS (2020). Yield prediction of Chinese cabbage (Brassica rapa var. glabra Regel.) using narrowband hyperspectral imagery and effective accumulated temperature. J. Agric. Life Sci..

[21] Kim DW (2018). Modeling and testing of growth status for Chinese cabbage and white radish with UAV-based RGB imagery. Remote Sens. Basel.

[22] Redmon, J., Divvala, S., Girshick, R. & Farhadi, A. You only look once: Unified, real-time object detection. In *Proceedings of the IEEE Computer Society Conference on Computer Vision and Pattern Recognition* (2016).

[23] PIX4D. Professional photogrammetry and drone mapping software |Pix4D. *PIX4D. *https://www.pix4d.com/ (2021).

[24] Esri Inc. ArcGIS Pro (Version 3.0.3). *Esri Inc.* https://www.esri.com/en-us/home (2023).

[25] Hunter JD (2007). Matplotlib: A 2D graphics environment. Comput Sci Eng.

[26] Phillips, D. *Python3 Object-oriented Programming, vol. 58* 12. 10.1109/TGRS.2004.834800 (2014).

[27] Chandrashekar G, Sahin F (2014). A survey on feature selection methods. Comput. Electr. Eng..

[28] Wickham H (2016). ggplot2: Elegant Graphics for Data Analysis.

[29] R Core Team. *R: A Language and Environment for Statistical Computing*. https://www.R-project.org/ (2021).

[30] Zhang J (2022). Multispectral drone imagery and SRGAN for rapid phenotypic mapping of individual chinese cabbage plants. Plant Phenom..

[31] Pantazi E (2022). Assessment of different object detectors for the maturity level classification of broccoli crops using UAV imagery. Remote Sens. Basel.

[32] Fu H, Wang C, Cui G, She W, Zhao L (2021). Ramie yield estimation based on UAV RGB images. Sens. Switzerl..

[33] Sun XX (2019). Genome-wide transcriptome analysis reveals molecular pathways involved in leafy head formation of Chinese cabbage (*Brassica rapa*). Hortic. Res..

[34] Wang H (2021). EasyIDP: A python package for intermediate data processing in UAV-based plant phenotyping. Remote Sens. Basel.

[35] Paszke, A. *et al.**PyTorch: An Imperative Style, High-Performance Deep Learning Library. *https://pytorch.org/ (2019).

[36] Dwyer, B. & Nelson, J. Roboflow (Version 1.0) [Software]. https://roboflow.com/ (2022).

[37] Bradski, G. The OpenCV Library. *Dr. Dobb’s Journal of Software Tools.* https://opencv.org/ (2000).

[38] Jordahl, K. GeoPandas: Python tools for geographic data. https://github.com/geopandas/geopandas (2014).

[39] QGIS Development Team. QGIS Geographic Information System. http://qgis.org (2009).

[40] Sokolova M, Lapalme G (2009). A systematic analysis of performance measures for classification tasks. Inf. Process Manag..

[41] Kuglin CD, Hines DC (1975). The phase correlation image alignment method. IEEE Int. Conf. Cybern. Soc..

[42] Guizar-Sicairos M, Thurman ST, Fienup JR (2008). Efficient subpixel image registration algorithms. Opt. Lett..

[43] Hoyer S, Hamman J (2017). xarray: N-D labeled arrays and datasets in Python. J. Open Res. Softw..

[44] Tucker CJ (1979). Red and photographic infrared linear combinations for monitoring vegetation. Remote Sens. Environ..

[45] Bendig J (2015). Combining UAV-based plant height from crop surface models, visible, and near infrared vegetation indices for biomass monitoring in barley. Int. J. Appl. Earth Observ. Geoinf..

[46] Gitelson AA, Gritz Y, Merzlyak MN (2003). Relationships between leaf chlorophyll content and spectral reflectance and algorithms for non-destructive chlorophyll assessment in higher plant leaves. J. Plant Physiol..

[47] Maccioni A, Agati G, Mazzinghi P (2001). New vegetation indices for remote measurement of chlorophylls based on leaf directional reflectance spectra. J. Photochem. Photobiol. B.

[48] Rouse, J. W., Haas, R. H., Schell, J. A. & Deering, D. W. Monitoring vegetation systems in the great plains with ERTS. In *Third Earth Resources Technology Satellite-1 Symposium, vol. 1* (1973).

[49] Gitelson A, Merzlyak MN (1994). Quantitative estimation of chlorophyll-a using reflectance spectra: Experiments with autumn chestnut and maple leaves. J. Photochem. Photobiol. B.

[50] Huete AR (1988). A soil-adjusted vegetation index (SAVI). Remote Sens. Environ..

[51] Hum YC, Lai KW, Mohamad Salim MI (2014). Multiobjectives bihistogram equalization for image contrast enhancement. Complexity.

[52] Harris CR (2020). Array programming with NumPy. Nature.

[53] Virtanen P (2020). SciPy 1.0: Fundamental algorithms for scientific computing in Python. Nat. Methods.

[54] Pedregosa F (2011). Scikit-learn: Machine learning in Python. J. Mach. Learn. Res..

