# Supplementary material for: UAV-based individual Chinese cabbage weight prediction using multi-temporal data
Source: Sci Rep. 2023 Nov 17;13:20122. doi: 10.1038/s41598-023-47431-y (PMC10656565; doi:10.1038/s41598-023-47431-y)
Supplement: Supplementary file 1 — Supplementary Information. [file 41598_2023_47431_MOESM1_ESM.docx]

UAV-based individual Chinese cabbage weight prediction using multi-temporal data

Andrés Aguilar Ariza, Masanori Ishii, Toshio Miyazaki, Aika Saito, Hlaing Phyoe Khaing, Hnin Wint Phoo, Tomohiro Kondo, Toru Fujiwara, Wei Guo, Takehiro Kamiya

**Supplementary tables**

**Table S1.** Flight dates.

| Time point (TP) | Flight Date | Days after transplanting (DAT) |
| --- | --- | --- |
| 1 | 2021-09-22 | 12 |
| 2 | 2021-09-29 | 19 |
| 3 | 2021-10-04 | 24 |
| 4 | 2021-10-08 | 28 |
| 5 | 2021-10-11 | 31 |
| 6 | 2021-10-14 | 34 |
| 7 | 2021-10-18 | 38 |
| 8 | 2021-10-20 | 40 |
| 9 | 2021-10-21 | 41 |
| 10 | 2021-10-25 | 45 |
| 11 | 2021-10-28 | 48 |
| 12 | 2021-11-01 | 52 |
| 13 | 2021-11-04 | 55 |
| 14 | 2021-11-05 | 56 |
| 15 | 2021-11-08 | 59 |
| 16 | 2021-11-10 | 61 |
| 17 | 2021-11-12 | 63 |
| 18 | 2021-11-15 | 66 |
| 19 | 2021-11-17 | 68 |
| 20 | 2021-11-19 | 70 |
| 21 | 2021-11-24 | 75 |
| 22 | 2021-11-26 | 77 |
| 23 | 2021-11-29 | 80 |
| 24 | 2021-12-06 | 87 |
| 25 | 2021-12-15 | 96 |
| 26 | 2021-12-20 | 101 |

**Table S2.** List of the variables used in the models.

| Level | Feature | Meaning | Source |
| --- | --- | --- | --- |
| First | red-RGB | Red channel | DJI Phantom 4 RTK |
| First | green-RGB | Green channel | DJI Phantom 4 RTK |
| First | blue-RGB | Blue channel | DJI Phantom 4 RTK |
| First | red-MS | Red channel | DJI P4 Multispectral |
| First | green-MS | Green channel | DJI P4 Multispectral |
| First | blue-MS | Blue channel | DJI P4 Multispectral |
| First | NIR | Near-infrared channel | DJI P4 Multispectral |
| First | red-edge | Red-edge channel | DJI P4 Multispectral |
| Second | RGBVI_RGB | Red-green-blue vegetation index computed from RGB | DJI Phantom 4 RTK |
| Second | GRVI_RGB | Green and red ratio vegetation index computed from RGB | DJI Phantom 4 RTK |
| Second | RGBVI | Red-green-blue vegetation index computed from MS | DJI P4 Multispectral |
| Second | NDVI | Normalized difference vegetation index | DJI P4 Multispectral |
| Second | NDR | Normalized difference red-edge index | DJI P4 Multispectral |
| Second | GNDVI | Green normalized difference vegetation index | DJI P4 Multispectral |
| Second | SAVI | Soil-adjusted vegetation index | DJI P4 Multispectral |
| Third | PH | Plant Height (cm) | DJI Phantom 4 RTK |
| Third | leaf angle | Leaf angle from the center to each height pixel value (°) | DJI Phantom 4 RTK |
| Third | volume | Plant volume (cm³) | DJI Phantom 4 RTK |

**Table S3.** The hyperparameters values used for finding the best training configuration for each regressor model.

| **Model** | **Tuning hyperparameters** | **Values** | |
| --- | --- | --- | --- |
|  |  | **min** | **max** |
| Partial Least Square | Number of components | 1.00 | 20 |
| Ridge | Regularization strength (L2) | -4 | -0.5 |
| Lasso | Regularization strength (L1) | -4 | -0.5 |
| Support Vector Machine-Linear  (SVM-Linear) | Kernel coefficient (gamma) | 0.0001 | 0.1 |
|  | Regularization parameter | 0.1 | 1000 |
| Random Forest (RF) | Number of trees | 300 |  |
|  | number of features required to split | 0.15 | 0.6 |
|  | Maximum depth of the tree | 2 | 32 |
|  | Number of samples required to split in the internal node | 2 | 8 |
|  | Maximum number of samples used to train each base estimator | 0.7 | 0.9 |

**Supplementary figures**


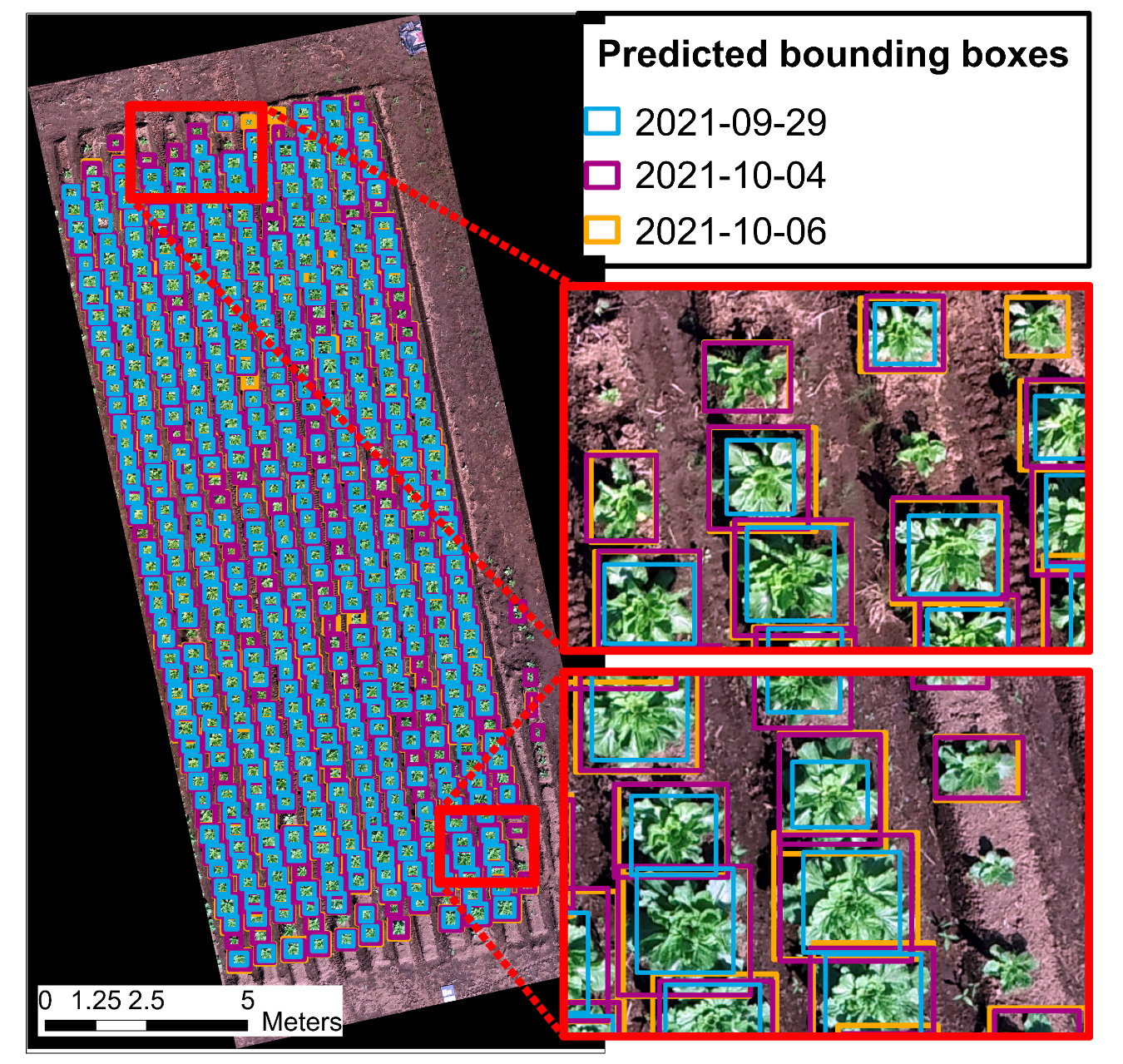


**Figure S1.** YOLO's bounding box predictions from three RGB orthomosaic imagery were acquired at three different time points in 2021 (September 29, October 4, and October 6). The enlarged image shows the predicted bounding boxes on the half of the field designated as the test. The colors indicate the bounding box prediction obtained at each time point. The figure was created with ArcGIS Pro version 3.0.3 [1].


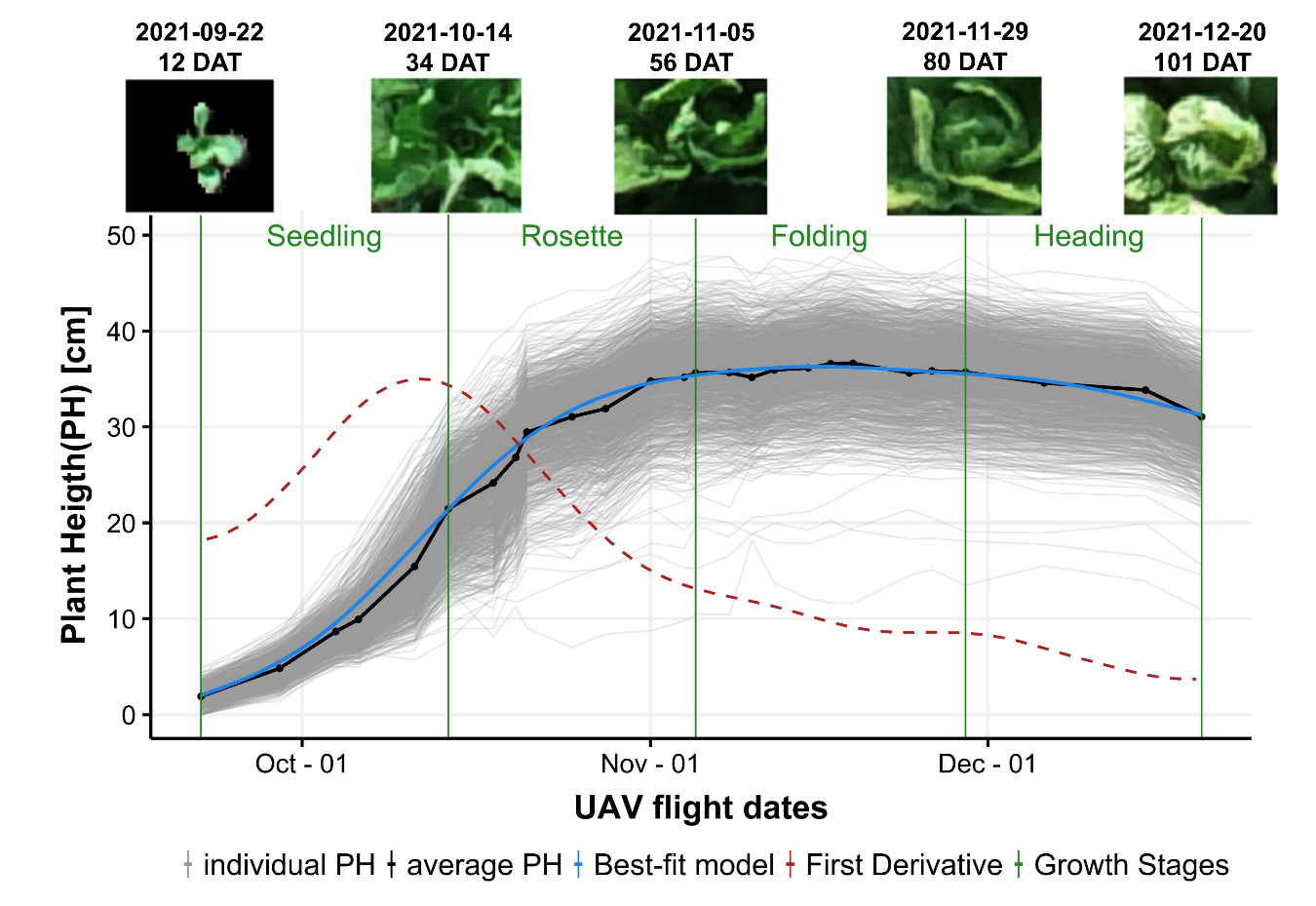


**Figure S2.** Plant height (PH) development of Chinese cabbage throughout their growth cycle. The cycle was divided into four stages (seedling, rosette, folding, and heading). The mean PH was calculated from 1,136 detected plants and is represented by the black line. A general additive linear model was fitted to the mean curve, shown by the blue line. The red dashed line represents the first derivative of the fitted model, which was used to identify changes in the growth pattern. Box plots were created with the ggplot2 package in R version 4.1.1 [2,3].


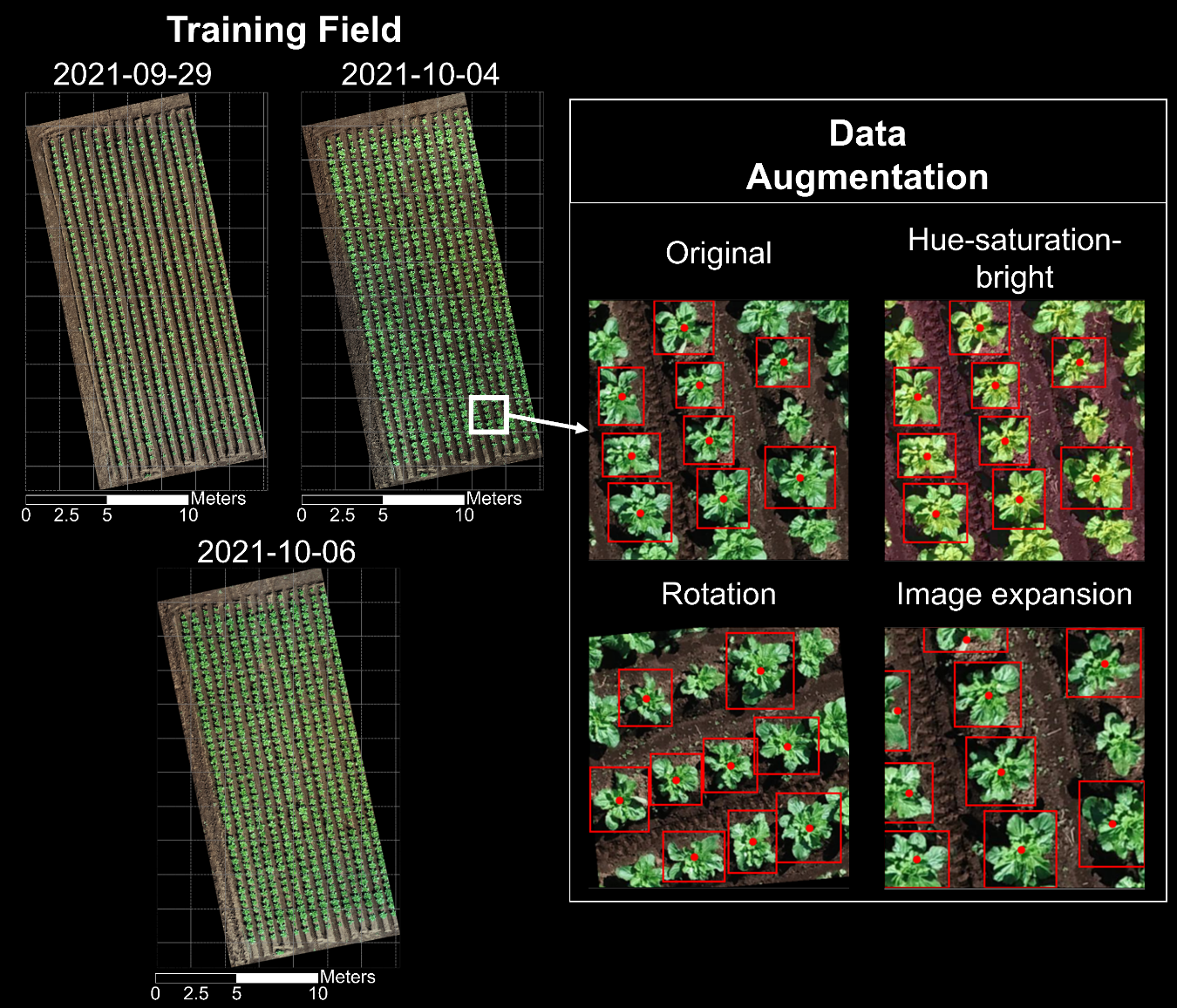


**Figure S3.** The left panel shows the RGB orthomosaic imagery acquired at three TPs (September 9, October 4, and October 6) used as a training dataset for the object detection model. The gray rectangles are the tiles (size 512 × 512 pixels; 2.08 × 2.08 m) in which the field was split. The right panel shows an example of an RGB image tile after being transformed using three augmentation functions. The red squares represent manually drawn bounding boxes for each plant, and the red dot denotes the center of the bounding box. Orthomosaic imagery was processed with Pix4D Mapper (PIX4D, 2021). Data augmentation figure examples were created with the Matplotlib package in Python version 3.9 [4,5]





**Figure S4.** Spatial assessment of 3D point cloud data in X- and Y- dimensions. The assessment was performed using two metrics: correlation-coefficient and cross-correlation displacement. (A) An example of the comparison between RGB (RGB camera) and the 2D RGB (3D point cloud data) grayscale images. Scatter and cross-correlation plots were used to compare both image differences. Cross-correlation is a similarity metric, quantifying the displacement between the two images transformed into Fourier space. (B) Histogram of correlation coefficient and cross-correlation displacement for a randomly selected 100 plants. The maximum displacement in X and Y was 3.81 mm; the median correlation-coefficient was 0.89. Figures created with the Matplotlib package in Python version 3.9 [4,5]


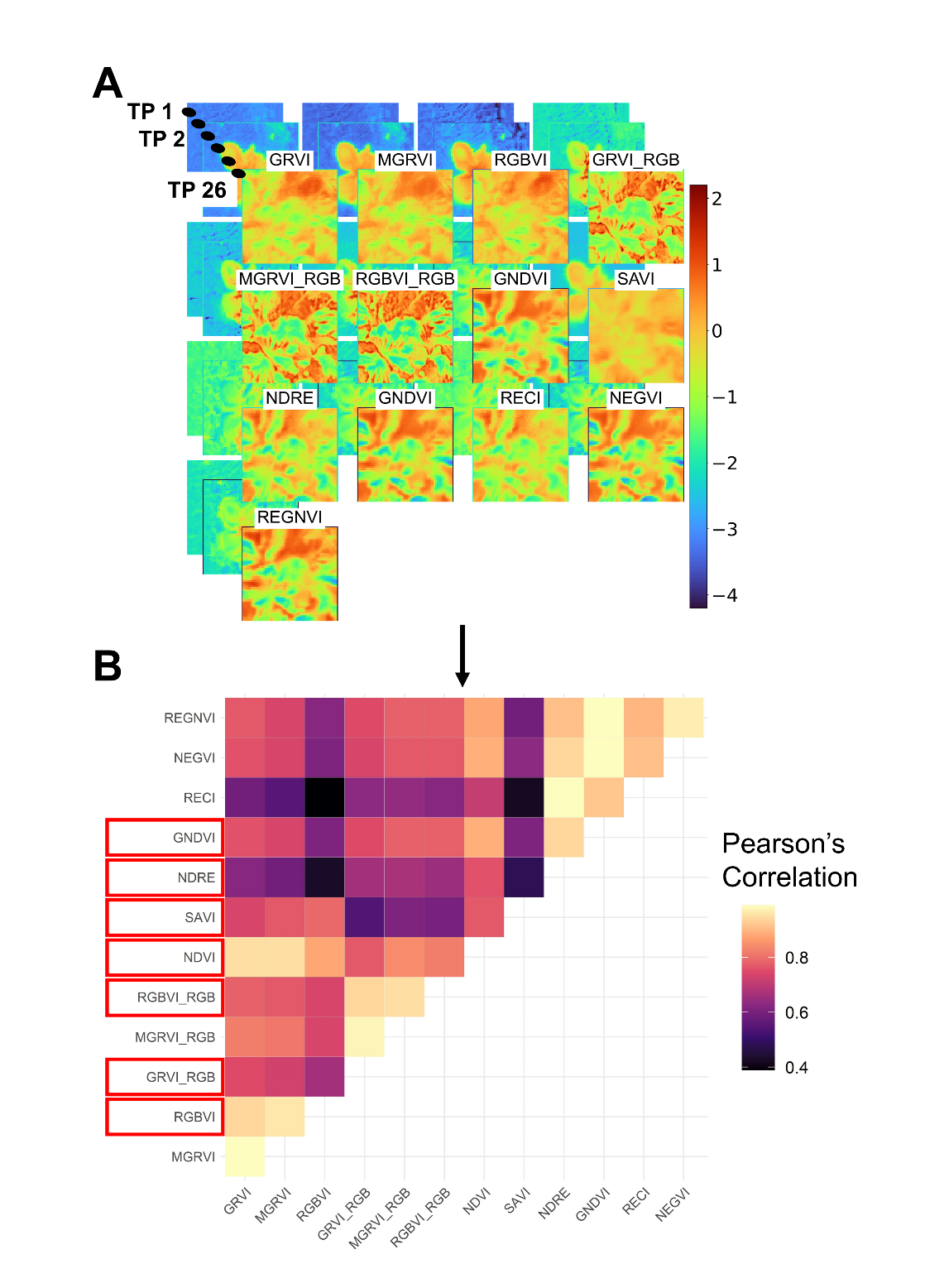


**Figure S5.** Pearson correlation among vegetation indices (VIs). (A) An example of the standardized VI data for one single plant throughout the 26 TPs. The figure was created with the Matplotlib package in Python version 3.9 [4,5]. These data were standardized before applying the correlation matrix (B). The seven selected VI traits are highlighted with a red square. The correlation plot was created with the ggplot2 package in R version 4.1.1 [2,3].


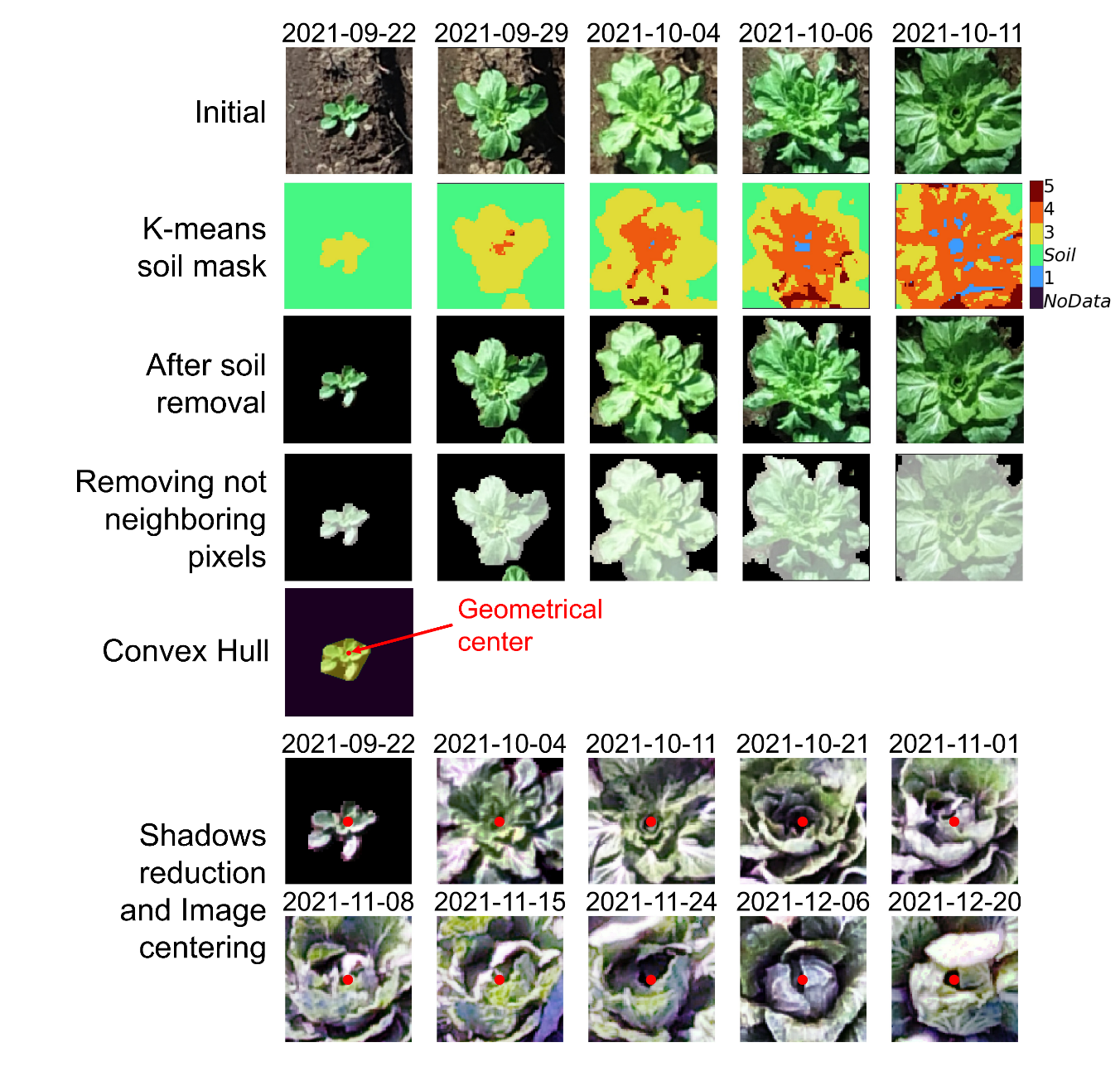


**Figure S6.** The image illustrates the different processing steps that were applied to an individual plant image with five TPs. The bottom panel displays the resulting individual plant image RGB with 10 TPs after histogram equalization and image center shift (red dot). The individual plant image examples were visualized with the Matplotlib package in Python version 3.9 [4,5].


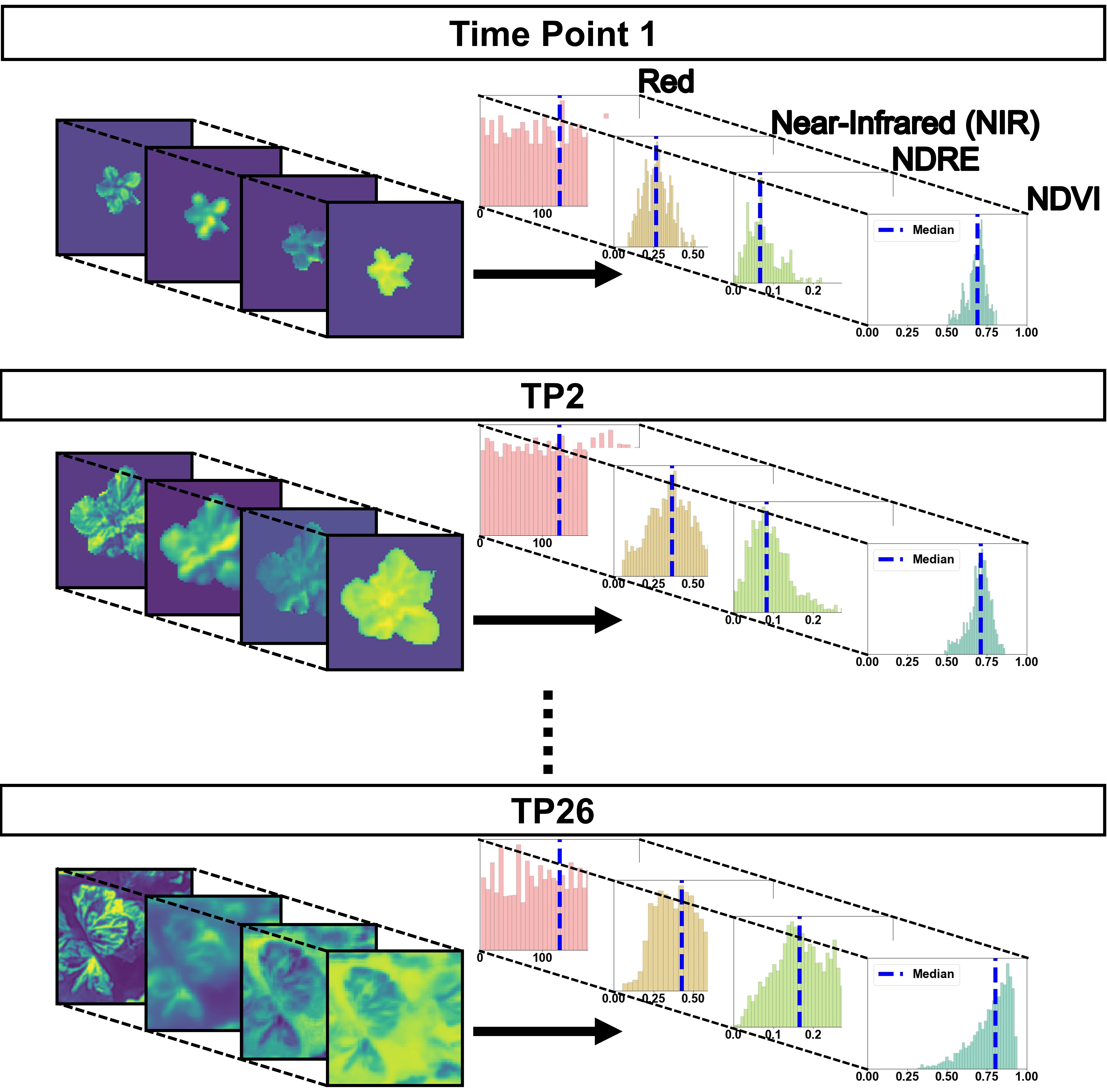


**Figure S7.** Individual plant features calculation. The figure shows the process of extracting multi-temporal features for four UAV-based data (e.g., NDVI, NDRE, NIR, and red). Left: images corresponding to each spectral and vegetation indices. Right: histogram for each image with the median indicated by the blue dashed line. The individual plant image examples were visualized with the Matplotlib package in Python version 3.9 [4,5].


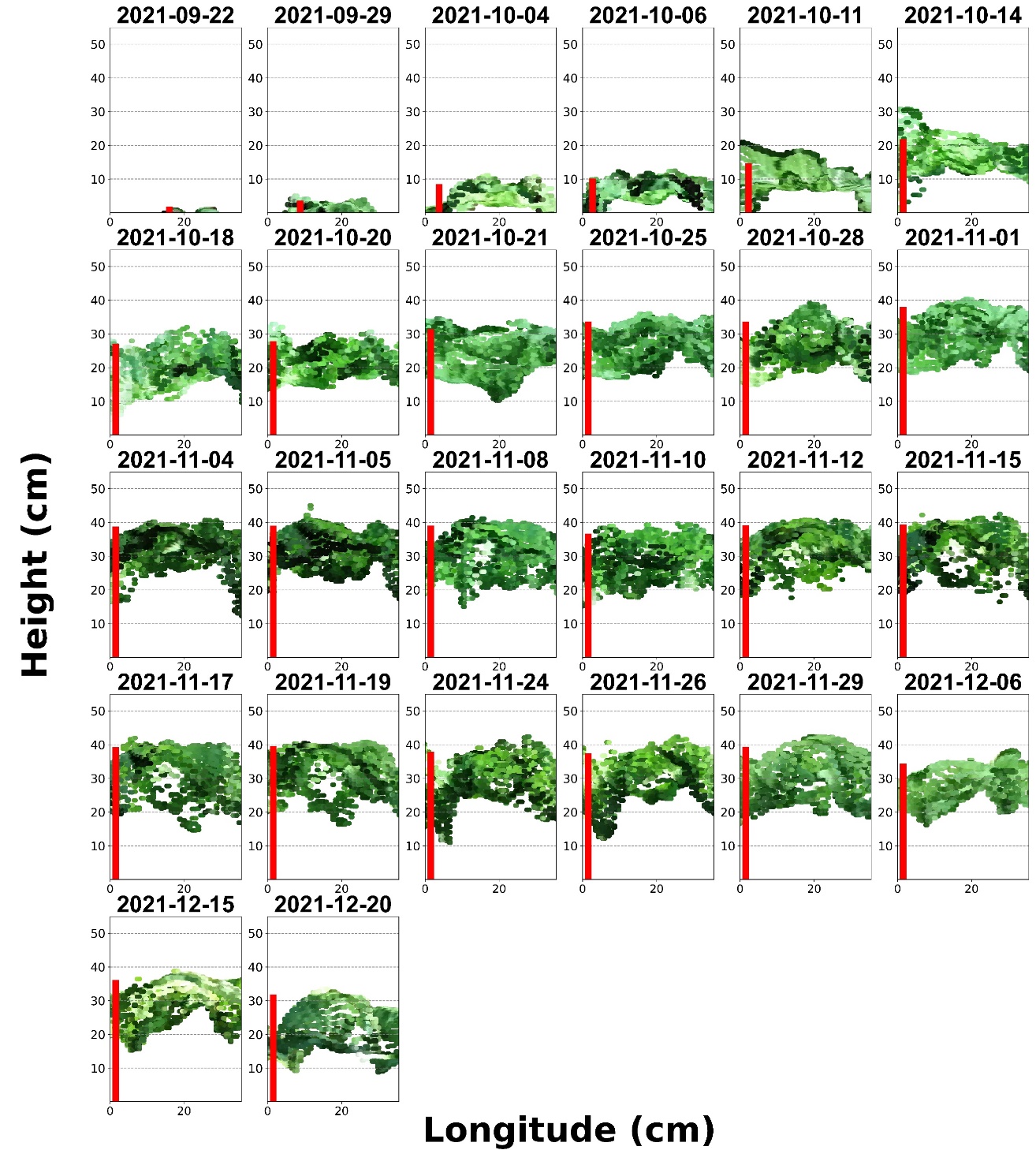


**Figure S8.** An example of an individual plant height value across the 26 TPs. The height values (represented with the red line) were extracted after transforming the 3D cloud point data into the plant height image. The red line represents the height from the ground to the 90% distribution of the plant height image, which is defined as plant height (PH). The PH figures were created with the Matplotlib package in Python version 3.9 [4,5].

**References**

1. Esri Inc. ArcGIS Pro (Version 3.0.3). *Esri Inc.* Preprint at (2023).

2. Wickham, H. *ggplot2: Elegant Graphics for Data Analysis*. (Springer-Verlag New York, 2016).

3. R Core Team. R: A Language and Environment for Statistical Computing. Preprint at https://www.R-project.org/ (2021).

4. Hunter, J. D. Matplotlib: A 2D graphics environment. *Comput Sci Eng* **9**, 3; 10.1109/MCSE.2007.55 (2007).

5. Phillips, D. *Python3 Object-oriented Programming*. vol. 58 12; 10.1109/TGRS.2004.834800 (2014).
